# Predicting long-term prognosis after percutaneous coronary intervention in patients with new onset ST-elevation myocardial infarction: development and external validation of a nomogram model

**DOI:** 10.1186/s12933-023-01820-9

**Published:** 2023-04-13

**Authors:** Zongwei Ye, Yanan Xu, Long Tang, Min Wu, Bing Wu, Tongjian Zhu, Jun Wang

**Affiliations:** 1 Department of Cardiology, Suzhou Ninth People’s Hospital, Soochow University, Suzhou, Jiangsu Province 215200 China; 2 Department of Cardiology, Pulmonary and Critical Care Medicine, The First Affiliated Hospital of Bengbu Medical College, Bengbu, Anhui China; 3 Department of Cardiology, People’s Hospital of Xuancheng City, The Affiliated Xuancheng Hospital of Wannan Medical College, Anhui, 242000 China; 4 Department of Oncology, Third People’s Hospital of Honghe Prefecture, Gejiu, Yunnan Province China; 5 Institute of Clinical Medicine, Department of Cardiology, Renmin Hospital, Hubei University of Medicine, Shiyan, Hubei 442000 China; 6 Department of Cardiology, Institute of Cardiovascular Diseases, Xiangyang Central Hospital, Affiliated Hospital of Hubei University of Arts and Science, Xiangyang, Hubei China

**Keywords:** ST-elevation myocardial infarction, Percutaneous coronary intervention, Major adverse cardiovascular events, Prediction nomogram, Triglyceride glucose index

## Abstract

**Background:**

The triglyceride glucose (TyG) index is a well-established biomarker for insulin resistance (IR) that shows correlation with poor outcomes in patients with coronary artery disease. We aimed to integrate the TyG index with clinical data in a prediction nomogram for the long-term prognosis of new onset ST-elevation myocardial infarction (STEMI) following primary percutaneous coronary intervention (PCI) .

**Methods:**

This retrospective study included new-onset STEMI patients admitted at two heart centers for emergency PCI from December 2015 to March 2018 in development and independent validation cohorts. Potential risk factors were screened applying least absolute shrinkage and selection operator (LASSO) regression. Multiple Cox regression was employed to identify independent risk factors for prediction nomogram construction. Nomogram performance was assessed based on receiver operating characteristic curve analysis, calibration curves, Harrell’s C-index and decision curve analysis (DCA).

**Results:**

In total, 404 patients were assigned to the development cohort and 169 to the independent validation cohort. The constructed nomogram included four clinical variables: age, diabetes mellitus, current smoking, and TyG index. The Harrell’s C-index values for the nomogram were 0.772 (95% confidence interval [CI]: 0.721–0.823) in the development cohort and 0.736 (95%CI: 0.656–0.816) in the independent validation cohort. Significant correlation was found between the predicted and actual outcomes in both cohorts, indicating that the nomogram is well calibrated. DCA confirmed the clinical value of the development prediction nomogram.

**Conclusions:**

Our validated prediction nomogram based on the TyG index and electronic health records data was shown to provide accurate and reliable discrimination of new-onset STEMI patients at high- and low-risk for major adverse cardiac events at 2, 3 and 5 years following emergency PCI.

**Supplementary Information:**

The online version contains supplementary material available at 10.1186/s12933-023-01820-9.

## Introduction

Acute myocardial infarction (AMI) is the cause of a major disease burden worldwide, and many known challenges exist in the management of AMI [1–2]. A significant portion of the patients who present to the emergency department exhibit symptoms that may be suggestive of AMI, making this a major condition encountered in daily clinical practice. Currently, percutaneous coronary intervention (PCI) is the standard of care employed to relieve myocardial ischemia resulting from the target stenosis, but accumulating evidence indicates that as many as 20% of AMI patients have a worse prognosis after successful PCI [3–6]. Therefore, the identification of potentially modifiable and residual risk factors would be helpful for AMI patients at high risk for a poor prognosis after stent implantation. Moreover, precise and early identification of patients at low risk for AMI based on such risk factors would be of profound clinical importance for fast initiation of individual comprehensive therapy and risk factor management [7].

Recent research has established that insulin resistance (IR) can be quantified using the triglyceride-glucose (TyG) index, an alternative biomarker that is both accessible and reliable [8]. Additionally, substantial statistical evidence indicates that the TyG index correlates with the progression of acute coronary syndromes (ACS) [8–12]. Furthermore, the TyG index has been shown to be a valid and effective prognostic factor in patients with ACS, with research showing that the TyG index can be used for risk stratification of ACS patients [8–12]. The molecular mechanisms underlying the predictive role of the TyG in coronary atherosclerosis have also been studied [8].

The pathology of IR is characterized by a complex interplay among factors such as metabolic disturbance, endothelial dysfunction, exaggerated inflammatory response, and smooth muscle cell dysfunction. These factors collectively contribute to the dysregulation of glucose metabolism and may increase the risk of various adverse outcomes [8]. Moreover, clinical data from direct comparisons indicate that the TyG index is more accurate than the homeostasis model assessment estimated insulin resistance (HOMA-IR) index and, thus, could provide incremental prognostic information for clinical outcomes in patients with coronary artery disease (CAD) [8, 13−14]. In addition, because the TyG index incorporates two metabolic indicators, fasting blood-glucose (FBG) and fasting triglyceride (TG), that can be readily measured, this biomarker offers the benefits of a low cost-to-benefit ratio, common availability in clinical practice, and being easily generalized for practical utilization.

Given that AMI is a complex disorder involving multiple pathophysiological mechanisms, binary diagnosis of coronary artery disease does not reflect the complexity of AMI or cardiovascular risk stratification [7]. Hence, a quantitative model for determining the risk of ACS based on multi-modal data is needed. However, the effectiveness of integrating the TyG index with electronic health records (EHR) data in a prediction model that can provide individualized risk stratification for ACS patients has not been determined. Therefore, we investigated the long-term prognostic value of the TyG index combined with EHR data among patients with new-onset ST-elevation myocardial infarction (STEMI) after PCI.

## Methods

### Study design and participants

This retrospective study used data from two centers to develop and validate a nomogram for predicting outcomes after PCI. We screened cases of new-onset STEMI without previously known CAD who were treated with PCI between December 2015 and March 2018 (Supplementary materials, Appendix).

The inclusion criteria for the study were: new-onset STEMI without CAD and complete and successful revascularization upon treatment with PCI; prescription of standard treatment regimens after PCI according to established guidelines; regular follow-up in the clinic after discharge; and available baseline clinical data, biochemical test results, and clinical endpoint data [15]. We excluded STEMI patients who received PCI at baseline. Institutional review board approval was obtained from The Affiliated Xuancheng Hospital of Wannan Medical College (No. 2023-lw004-01) and Xiangyang Central Hospital (No.2023-023). The requirement to obtain informed patient consent for inclusion in this analysis was waived by the institutional review board.

### Definitions of cardiovascular risk factors

Patients who were taking any antihypertensive medication to control their blood pressure or with blood pressure over 140/90 mmHg measured on three or more separate occasions were defined as hypertension patients [16]. Patients with a diabetes history or whose measured fasting and/or postprandial blood glucose exceeded the standards set by guidelines were defined as diabetes patients [17]. Consumption of more than 30 g of alcohol per day was defined as current drinking.

### Biochemical tests

Prior to emergency coronary angiography, myocardial enzymonram evaluation as well as D-dimer measurement were performed. After PCI, fasting blood-glucose (FBG), lipid, and albumin levels were measured. The TyG index was calculated according to the following formula : ln [fasting triglyceride (mg/dl)*fasting glucose (mg/dl)]/2 [8].

### Cardiac ultrasound

A transthoracic echocardiography exam with color Doppler echocardiography was performed for the evaluated participants by a senior ultrasound physician after the procedure. The left ventricular end-diastolic diameter (LVEDD) was measured on standard M-mode echocardiography. The left ventricle ejection fraction (LVEF) was determined using the modified Simpson method.

### Coronary angiography and PCI

Upon patient admission for STEMI, the standard treatment protocol was followed, and coronary angiography was performed by an experienced senior cardiologist. For patients with new-onset STEMI, the first loading dose (aspirin plus clopidogrel ) was administered as soon as possible before primary PCI. An in-house team of physicians performed each PCI procedure according to the coronary anatomy and clinical condition of each evaluated patients. Successful and complete revascularization was defined as successful stent implantation with residual stenosis < 20% and TIMI (Thrombolysis in Myocardial Infarction) flow of grade 3 at the time of the procedure. Discontinuation of dual antiplatelet therapy was not recommended unless an indisputable reason for doing so was provided. All patients were advised to take aspirin (100 mg/day) indefinitely and a P2Y12 inhibitor (75 mg/day) after their index procedure for the recommended duration according to current guidelines [15].

### Clinical endpoint

Outpatient clinical visits or telephone interviews were conducted for clinical follow-up. The median follow-up duration among the study population was 51.71 months. The primary clinical endpoint of the study was the occurrence of major adverse cardiac events (MACEs), which refers to the composite of all-cause mortality, recurrence of myocardial infarction, in-stent restenosis, and revascularization.

### Statistical analysis

Statistical analyses of the study results were carried out using SPSS 26 and R software 4.2.2. Descriptive statistics are expressed as frequencies and proportions for categorical variables and as medians and interquartile ranges (IQRs) or means and standard deviations (SDs) for continuous variables. The distributions of putative variables between the development and external validation cohorts were compared using the Mann–Whitney U test (for continuous variables with non-normal distributions) and the exact Fisher test or χ^2^ test (for categorical variables). The median (95% confidence interval [CI]) incident of MACEs was analyzed with the Kaplan–Meier method. Differences in the incidence of MACEs between the development and independent validation cohorts were determined using the log-rank test. *P* values < 0.05 indicated a significant result for all comparisons. A multivariate Cox’s proportional hazards model applying the adaptive least absolute shrinkage and selection operator (LASSO) was employed, and hazard ratios (HRs) were calculated using Cox regression analyses with 95% CIs or *P*-values. The predictive performance of the prediction nomogram was evaluated using three methods [18]. First, its discriminatory ability was analyzed by determining the Harrell’s concordance index (C-index) and corresponding 95% CI. Second, the model’s calibration was represented by calibration plots predicting the probability of MACE risk at 2, 3 and 5 years versus the observed probability. Third, the nomogram was analyzed for its net benefit for different threshold probabilities by decision curve analysis (DCA). To determine these time points, the observed median incidence of MACEs was taken into account. An individual risk score was derived from the nomogram for patients in the development cohort. The optimal cutoff point for each model was calculated for stratification of patients into low-risk and high-risk categories. The log-rank statistic was applied to determine the optimal cutoff point to provide the largest discrepancy in MACE risk between the low-risk and high-risk groups.

## Results

### Patient characteristics

**Fig. 1:**
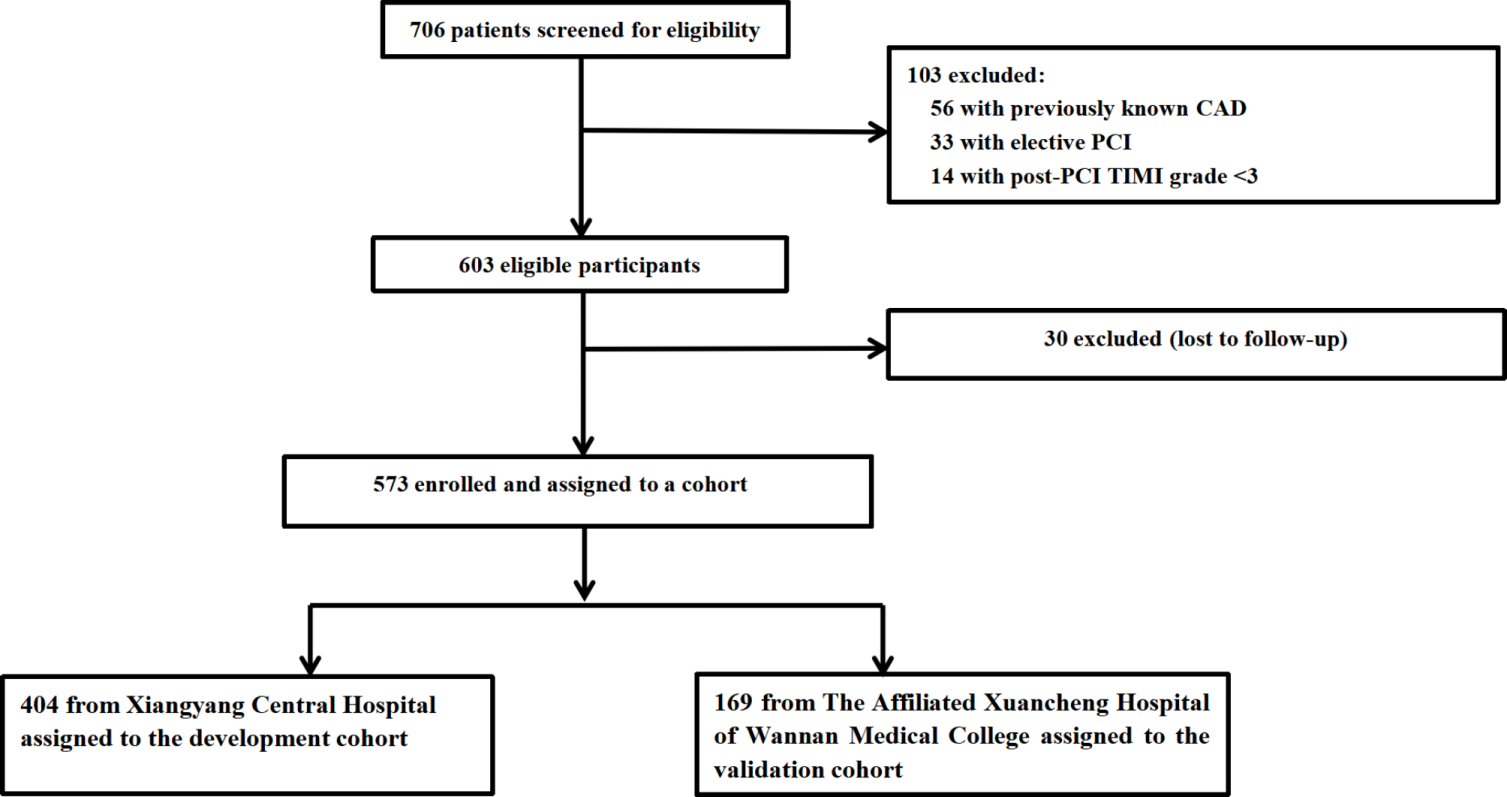
Inclusion of new onset ST-elevation myocardial infarction (STEMI) patients following primary percutaneous coronary intervention (PCI) (n = 706) and establishment of the development cohort (n = 404) and the external validation cohort (n = 169)

Retrospective screening identified 573 patients with new-onset STEMI who received emergency PCI between December 2015 and March 2018 and met the eligibility criteria for this study. These patients were included in the development cohort (n = 404; datasets from Xiangyang Central Hospital) and the independent validation cohort (n = 169; datasets from The Affiliated Xuancheng Hospital of Wannan Medical College) (Fig. 1).

Among all evaluated patients, the average age was 57.0 (48.0–67.0) years, and the majority of patients (81.8%) were male. In the development and external validation cohorts, 326 (80.7%) patients and 143 (84.6%) patients were men, respectively.

Over the median follow-up time of 51.71 months, 96 MACEs occurred (16.8%), with similar incidences in the development and external validation cohorts. The development and external validation cohorts were well-matched overall, with the exception of a significant difference in the incidence of hypertension (Table 1).

**Table 1:** Baseline patient characteristics and MACEs (clinical outcome) in the development and validation cohorts.^*,¶^

Characteristic or Outcome	All cohort (n = 573)	Development cohort (n = 404)	Validation cohort (n = 169)	*P*
Male sex	469 (81.8%)	326 (80.7%)	143 (84.6%)	0.321
Age, years	58.0 [49.0;68.0]	58.0 [48.0;68.0]	58.0 [49.0;68.0]	0.694
Hypertension	268 (46.8%)	177 (43.8%)	91 (53.8%)	0.035
Diabetes mellitus	146 (25.5%)	95 (23.5%)	51 (30.2%)	0.118
Current smoking	250 (43.6%)	180 (44.6%)	70 (41.4%)	0.550
Current drinking	124 (21.6%)	92 (22.8%)	32 (18.9%)	0.365
Family history of CAD	69 (12.0%)	51 (12.6%)	18 (10.7%)	0.602
Systolic blood pressure, mmHg	120 [109;133]	120 [109;132]	121 [110;134]	0.635
Diastolic blood pressure, mmHg	76.4 (12.6)	76.1 (12.3)	77.1 (13.4)	0.390
Heart rate, bpm	80.0 [72.0;91.0]	80.0 [71.0;91.0]	81.0 [75.0;91.0]	0.249
Body mass index, kg/m^2^	25.2 [23.4;27.7]	25.2 [23.3;27.6]	25.1 [23.4;27.8]	0.947
Left ventricular end-diastolic dimension, mm	50.0 [47.0;52.0]	50.0 [47.0;52.2]	50.0 [47.0;52.0]	0.832
Left ventricular ejection fraction	59.0 [55.0;63.0]	59.0 [55.0;62.0]	60.0 [56.0;63.0]	0.129
Creatine kinase, U/L	237 [102;856]	254 [102;834]	215 [102;856]	0.587
Creatine kinase-MB, U/L	28.0 [15.3;89.2]	27.6 [15.4;91.8]	28.4 [15.1;79.8]	0.942
D-dimer, mg/L	146 [91.0;281]	146 [93.0;294]	143 [89.0;260]	0.531
Albumin, mg/dL	40.0 [36.1;42.7]	39.9 [36.1;43.2]	40.2 [36.2;42.2]	0.865
Low-density lipoprotein cholesterol, mmol/L	2.96 [2.36;3.54]	3.01 [2.39;3.55]	2.88 [2.25;3.52]	0.174
Total cholesterol, mmol/L	4.65 [3.90;5.32]	4.66 [3.85;5.32]	4.61 [4.00;5.30]	0.965
Fasting blood glucose, mmol/L	8.06 [6.82;10.6]	8.05 [6.81;10.6]	8.06 [6.87;10.8]	0.589
Triglyceride, mmol/L	1.73 [1.13;2.87]	1.72 [1.08;2.90]	1.74 [1.22;2.78]	0.701
Triglyceride glucose (TyG) index	5.05 (0.40)	5.05 (0.40)	5.07 (0.39)	0.557
MACEs	96 (16.8%)	68 (16.8%)	28 (16.6%)	1.000
All-cause mortality	29 (5.06%)	21 (5.20%)	8 (4.73%)	0.982
Re-myocardial infarction	4 (0.70%)	3 (0.74%)	1 (0.59%)	1.000
In-stent restenosis	10 (1.75%)	6 (1.49%)	4 (2.37%)	0.491
Revascularization	64 (11.2%)	45 (11.1%)	19 (11.2%)	1.000

* Values are given as n (%), mean ± SD, or median (IQR)

¶ Here and below, all abbreviations are defined in the text

### Potential predictors of MACEs and construction of the nomogram

**Fig. 2:**
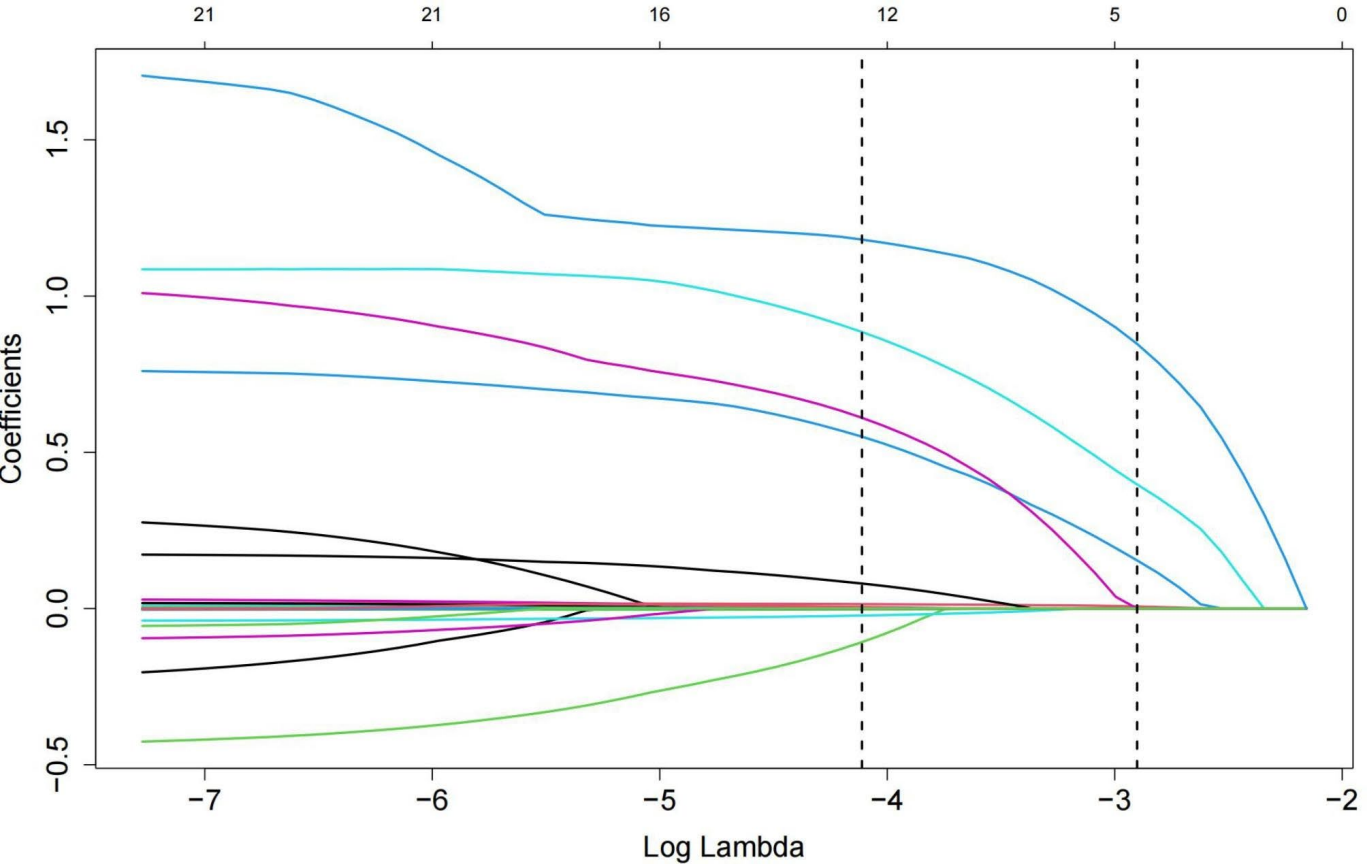
LASSO model coefficient trendlines of 22 variables for long-term prognosis. The abscissa represents the optimal tuning parameter λ, and the ordinate represents the regression coefficient

**Fig. 3:**
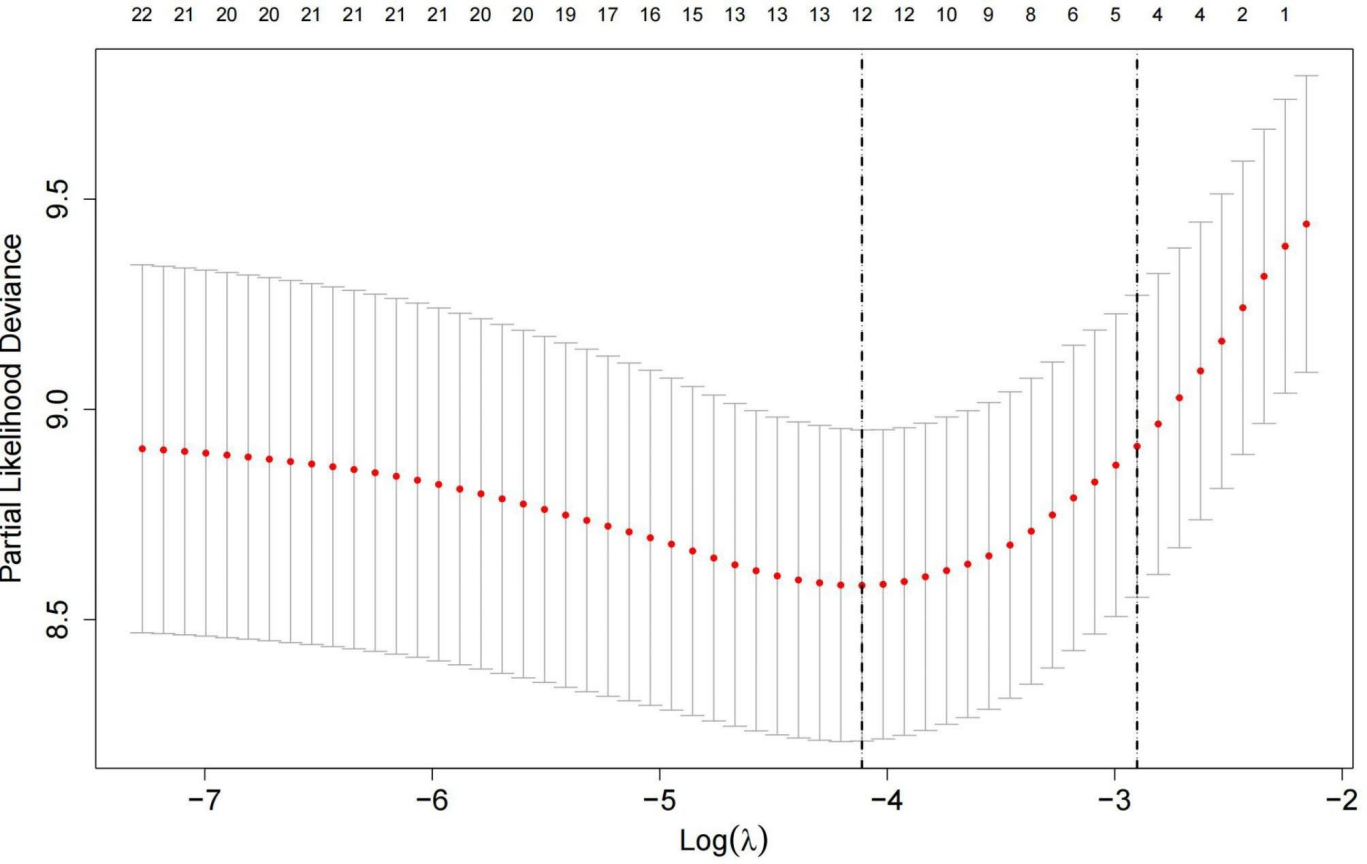
Tuning parameter (lambda, λ) selection cross-validation error curve. The abscissa represents the optimal tuning parameter λ, and the ordinate represents the binomial deviation (binomial deviance). Employing the optimized four nonzero coefficients derived by 10-fold cross-validation, the vertical line was drawn, and further multivariate Cox regression analysis was conducted

**Fig. 4:**
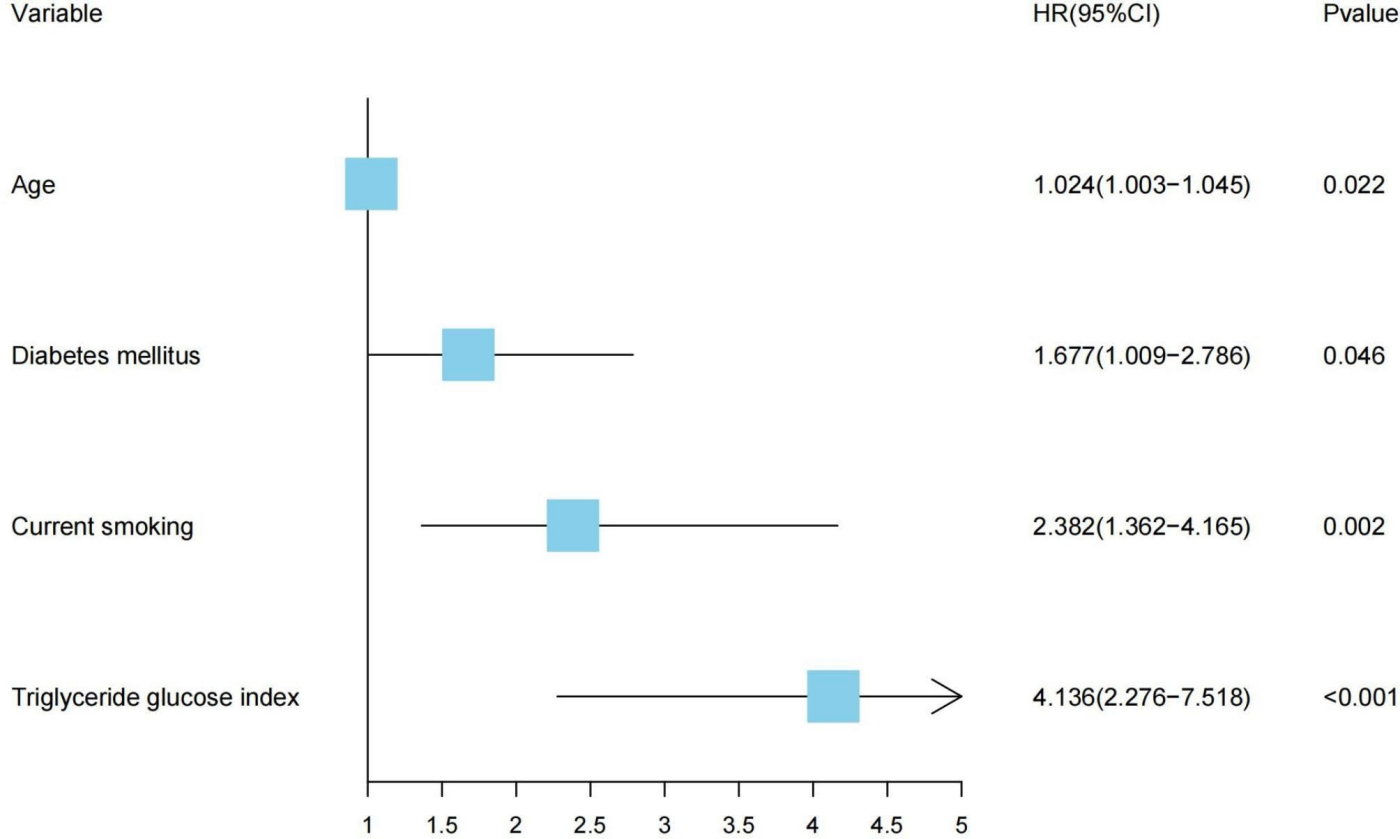
Forest plot of hazard ratios (HRs) for the independent prognostic variables identified by multivariate Cox regression in the development cohort

**Fig. 5:**
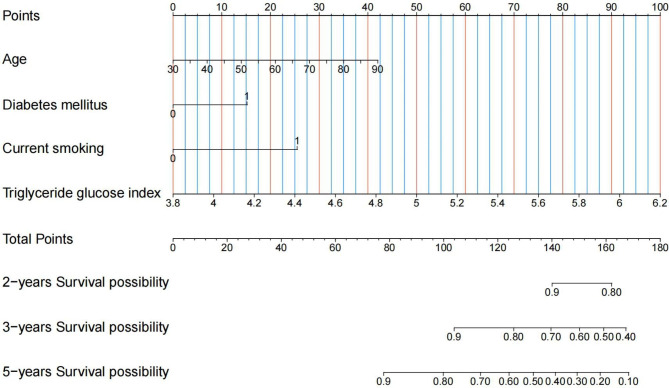
Prediction nomogram derived from multivariate Cox regression analysis. The vertical line is followed from the value of each clinical characteristic on the top row to assign points for each patient, and all six characteristics are added into a single matrix to obtain the point total (middle row). To calculate the probability of MACEs, a vertical line is drawn across the bottom two rows, applying the total points

Four variables were identified as potential predictors of MACEs based on nonzero coefficients from the LASSO regression model in the development cohort: age, diabetes mellitus, current smoking, and TyG index (Figs. 2 and 3). Multivariate Cox regression analysis further identified age (HR: 1.024, 95% CI: 1.003–1.045, *P* = 0.022), diabetes mellitus (HR: 1.677, 95% CI: 1.009–2.786, *P* = 0.046), current smoking (HR: 2.382, 95% CI: 1.362–4.165, *P* = 0.002), and TyG index (HR: 4.136, 95% CI: 2.276–7.518, *P* < 0.001) as independent risk factors for MACEs (Fig. 4). Subsequently, these four independent factors were implemented in the construction of the prediction nomogram for predicting MACE risk at 2, 3 and 5 years in new-onset STEMI patients treated with PCI (Fig. 5).

### Performance of the nomogram

**Fig. 6:**
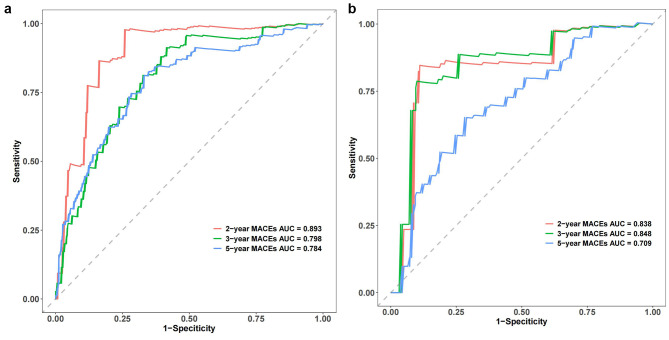
ROC curve analysis of the predictive accuracy of the nomogram for MACEs in the development (**A**) and independent validation cohorts (**B**)

We calculated the Harrell’s C-index and area under the curve (AUC) values to evaluate the discriminatory capacity of the prediction nomogram to identify new-onset STEMI patients treated with PCI at high risk for MACEs. The Harrell’s C-index for the ability of the nomogram to discriminate low- and high-risk cases in the development cohort was 0.772 (95% CI : 0.721–0.823). Implementation of the nomogram in the independent verification cohort yielded a similar Harrell’s C-index for the nomogram of 0.736 (95% CI: 0.656–0.816). Meanwhile, receiver operating characteristic (ROC) curve analysis determined that the AUC values for the ability of the nomogram to predict the 2-, 3- and 5-year MACE risks were 0.893, 0.798 and 0.784, respectively, in the development cohort, and the corresponding values were 0.838, 0.848 and 0.709, respectively, in the independent validation cohort (Fig. 6A and B).

**Fig. 7:**
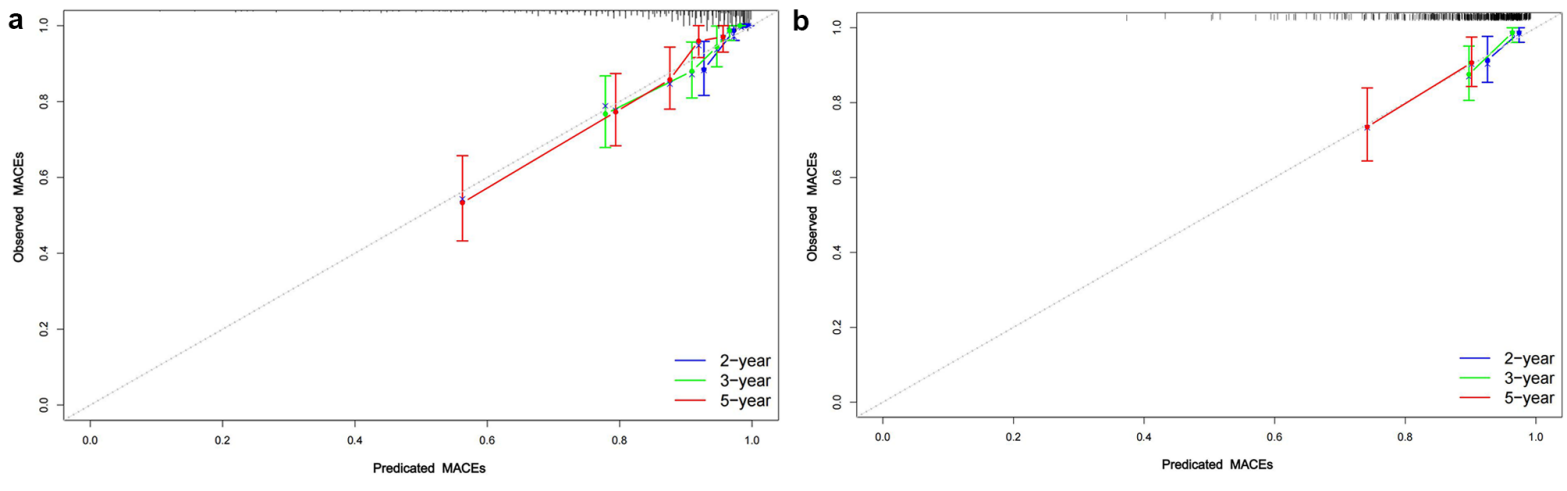
Calibration curves for MACE risk predictors in the development (**A**) and independent validation cohorts (**B**). Gray dotted line represents the ideal reference line where predicted probabilities would match the observed the risk possibility of MACEs. Thus, high-quality prediction of MACE risk is represented by the red line, and the black dashed line shows more precise predictions of long-term outcome

**Fig. 8:**
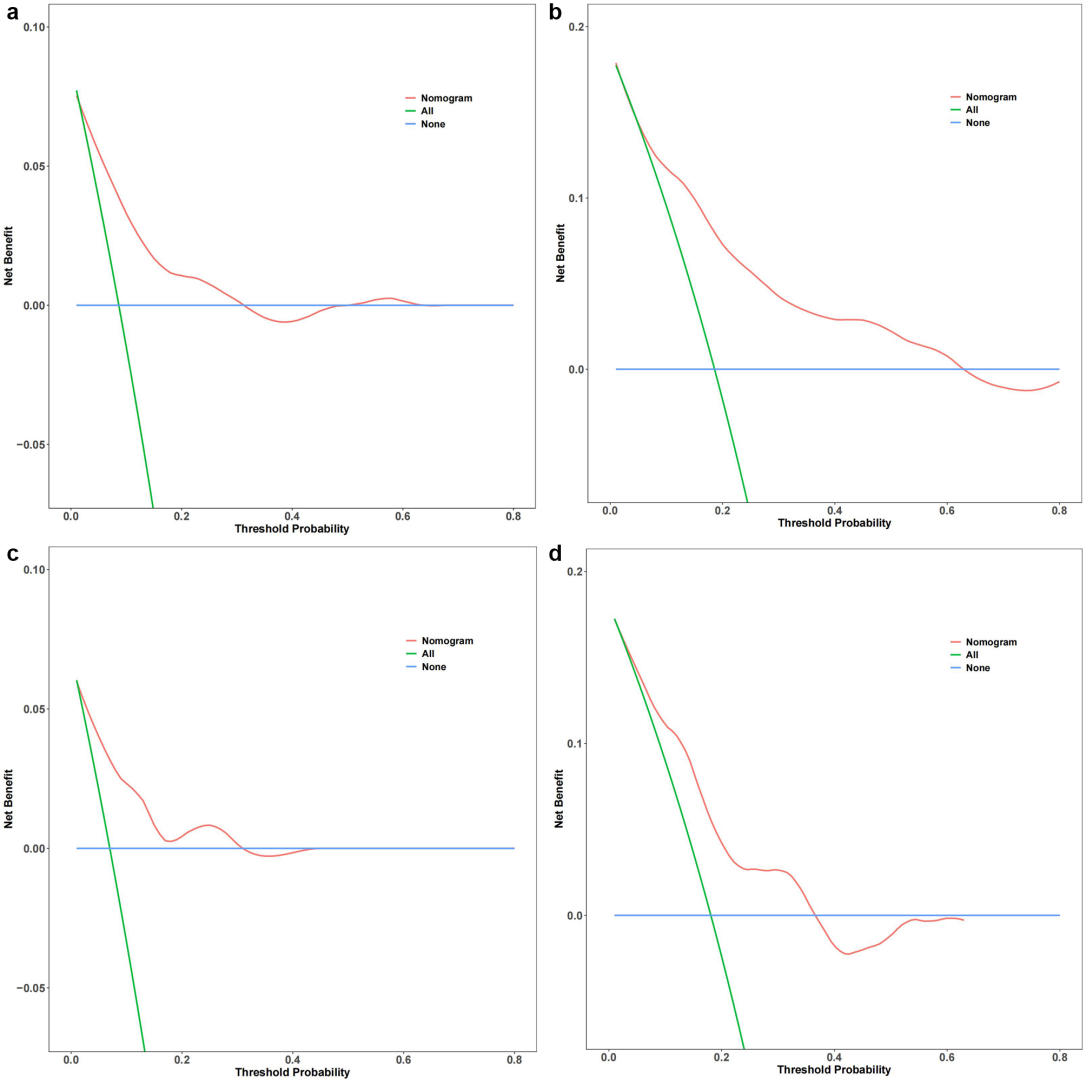
Decision curve analysis (DCA) for predicting 3-year MACE risk in the development (**A**) and independent validation cohorts (**C**) and the 5-year MACE risk in the development (**B**) and independent validation cohorts (**D**). The graph depicts the expected net benefit for each patient with respect to the risk for MACEs as predicted by the nomogram. With increasing model curve length, the net benefit increases

**Fig. 9:**
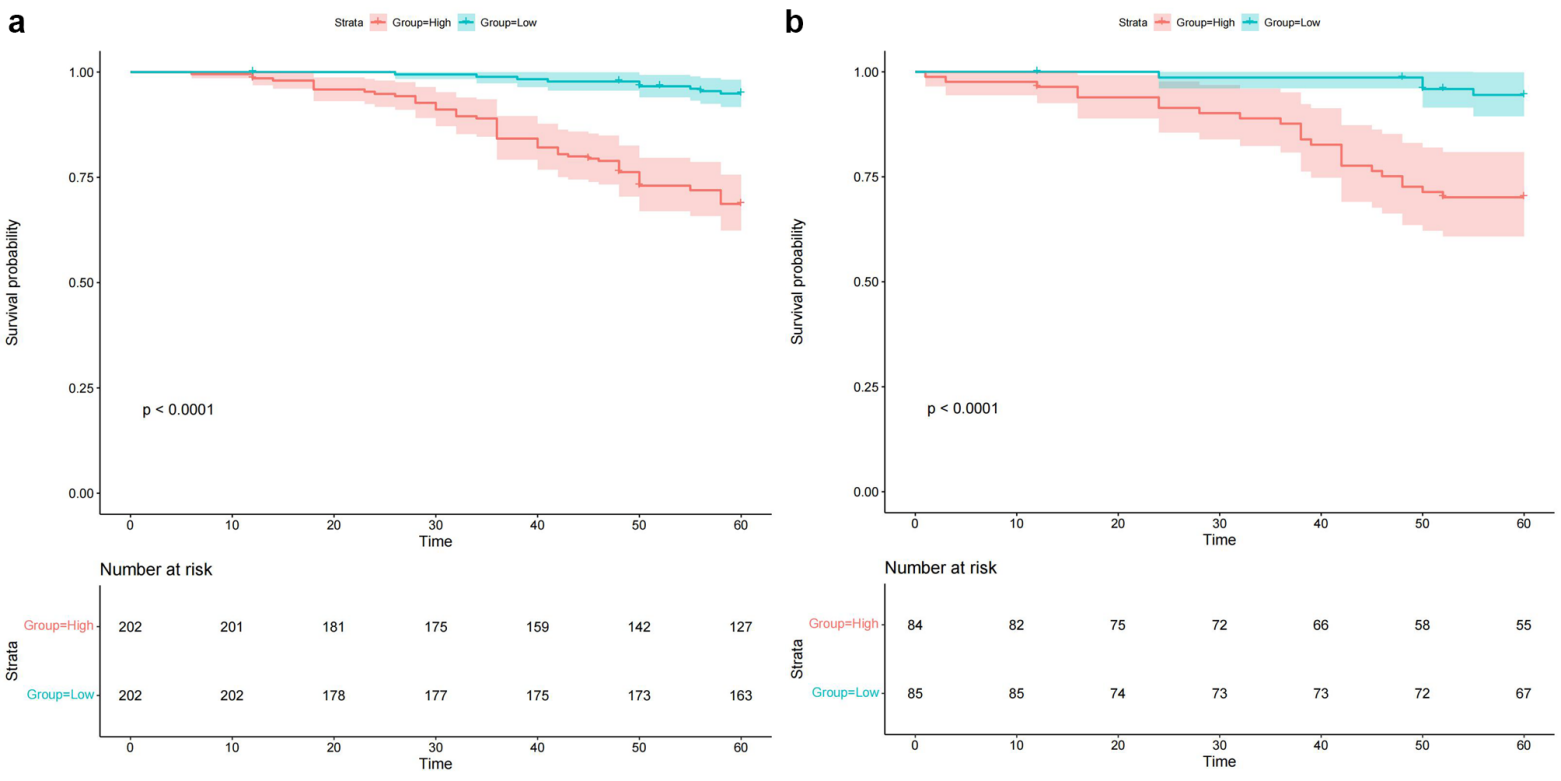
Cumulative MACE-free survival of patients in the development (**A**) and independent validation cohorts (**B**) stratified according to MACE risk (high risk vs. low risk) using the median nomogram score

Calibration plots were established to evaluate the consistency between the nomogram and observed probabilities for MACE risks for identifying patients with new-onset STEMI patients following PCI at risk for MACEs. From the calibration plots, the predicted probabilities from the nomogram in the development and independent validation cohorts were comparable to the observed probabilities for 2-, 3- and 5-year MACE risks (Fig. 7A and B). Furthermore, following an evaluation of the performance of the nomogram, DCA was also carried out, which also confirmed the predictive efficacy of the prediction nomogram. DCA exhibited that the threshold probability for prediction of 3-year MACE risk was 2–32% and that for 5-year MACE risk was 5–63% in the development cohort (Fig. 8A and B). Similarly, DCA in the independent validation cohort produced threshold probability ranges for the prediction nomogram of 3–33% for 3-year MACE risk and 5–36% for 5-year MACE risk (Fig. 8C and D). Additionally, our prediction nomogram performed well based on ROC curve analysis, Harrell’s C-index, calibration curves, and DCA. Patient stratification into two risk groups (high vs. low risk score) was performed by applying the median risk score in the development and independent validation cohorts (Fig. 9A and B). From Kaplan–Meier analysis, MACEs were more likely to occur in the high-risk groups than in the low-risk groups of both the development independent validation cohorts (both *P <* 0.001). Therefore, following the establishment of the prediction nomogram, patients with a high risk score had a propensity for poorer outcome compared with those with a low risk score.

## Discussion

Our prediction nomogram for the probability of MACEs in patients with new-onset STEMI at 2, 3 and 5 years after emergency PCI combines a biomarker of IR, the TyG index, with several traditional clinical risk factors (age, diabetes mellitus, and current smoking). This nomogram constructed in a development cohort was validated to be effective in an independent validation cohort according to AUC values, Harrell’s C-indexes, calibration curves and DCA. The good predictive performance of our prediction nomogram suggests that the variables included provide new insight into the risk stratification of STEMI, which could support the development of novel strategy.

Atherosclerosis is a dynamic process that evolves throughout the course of ACS and is promoted by multiple factors related to inflammation and the immune system, imbalance of the autonomic nervous system and metabolic disturbance [19–24]. Therefore, the occurrence, development and clinical outcome of ACS are influenced by the interactions of multiple systemic factors rather than any single factor. As noted elsewhere, Zheng et al. [25] constructed a simple-to-use nomogram, integrating three clinical features that are both straightforward to obtain and routinely collected in clinical practice, evaluating cardiac mortality in non-ST-segment elevation ACS patients after PCI. Consistently, emerging evidence demonstrates that a prediction nomogram considering five conventional clinical features that are easy to obtain in daily clinical work in cardiovascular risk assessment can provide clinicians with a non-invasive and simple method for assessing MACE risk in patients with ACS who are scheduled for emergency coronary angiography [26]. Undeniably, single-modality data alone cannot systematically and comprehensively depict the degree of coronary atherosclerosis in patients, especially for new-onset STEMI patients who have undergone emergency complete and successful revascularization. Therefore, approaches to understanding and managing ACS have evolved from focusing on “vulnerable plaques” to “vulnerable patients” who are suffering from an atheromatous-plaque burden, metabolic disturbance, as well as inflammatory and oxidative stress responses leading to plaque disruption to build a platform for better risk stratification for ACS patients [22]. Systematic ACS risk assessment is a process for identifying individuals who are at an increased risk of an adverse outcome, and such assessment could inform clinical decision-making by identifying high-risk patients who may require extra care and resources [22, 26–28]. Notably, residual cardiovascular risk is a persisting clinical problem that limits the benefit some patients can receive from the long-term success of PCI [26–27, 29−30]. Therefore, in the present study, a nomogram was developed for risk prediction in patients with STEMI treated with PCI that incorporates routine clinical data collected from EHR and a biomarker of metabolism, the TyG index.

The TyG index, which incorporates two major metabolism biomarker subtypes, is considered to be a representative biomarker of IR [8, 31]. Consistent clinical data have demonstrated that the TyG index offers prognostic efficacy in various cardiovascular and cerebrovascular diseases, including ACS, heart failure, atrial fibrillation and stroke [8, 32–33]. Emerging evidence demonstrates that a longitudinal correlation exists between the baseline TyG index and cardiovascular disease [34]. Another multi-center study found that a higher TyG index may identify patients with a high risk of multi-vessel CAD [35]. Additionally, the TyG index was shown to serve as a risk management strategy for CAD patients with different glucose metabolic states that can help determine the severity of CAD and facilitate healthcare decision-making [35]. A retrospective cohort study confirmed that an increased TyG index was independently related to a poor outcome in ACS patients with diabetes [36], and based on clinical data from 1282 diabetes patients with new-onset stable CAD over a long follow-up, Jin et al. observed a close correlation between the TyG index and adverse cardiovascular outcomes [37]. Our overall findings in the present study are in line with the previous literature indicating that the TyG index could benefit prediction strategies for long-term prognosis in ACS patients [8, 36]. However, previous studies included patients with a PCI history or prior MI that might affect the TyG index [36]. Therefore, in order to reduce the confounding effects of such factors in the current study, patients with previous CAD diagnoses were strictly excluded. Moreover, the benefit of incorporating the TyG with other clinical risk factors for risk stratification of patients had not been reported. Our prediction nomogram can play a valuable role in clinical work based on its ease of use in a wide variety of health care systems [18]. The results of the present study support the incremental prognostic value for long-term prognosis of adding the TyG index into a model that includes well-established risk factors among ACS patients [36–37]. Our prediction nomogram on the basis of the TyG index as a metabolism-related biomarker combined with EHR data offers a relatively straightforward clinical tool that can be obtained in little time using a simple intake form. Nevertheless, it is universally acknowledged that any prognostic model should only be accepted into clinical practice when it performs well and achieves the desired goals among researchers and physicians [18]. External validation, preferably in multiple, disparate datasets, is the “gold standard” and frequently utilized to determine whether a model performs well and should be incorporated into the clinical setting. In the present study, the development and independent validation cohorts were sourced from different centers of patients, and our external validation demonstrated that the prediction nomogram had satisfactory consistency for the prediction of MACEs. However, the prevalence of a strongly weighted prognostic risk factor, hypertension, was lower in the.

development cohort compared than in the external validation cohort. Nonetheless, our model performed well, displaying good discrimination as reported by C-index values of 0.772 for the development set and 0.736 for the validation set. Additionally, our model showed good performance based on calibration curves. The DCA also provided clinical evidence that the clinical accuracy of the prediction nomogram in both the development and external validation cohorts. Still, replication and further validation of our findings in other well-defined populations is necessary to ensure the robustness of our model.

Despite the close association between the TyG index and adverse cardiovascular outcomes, the underlying mechanism remains unclear. Pathologically, IR contributes to the interaction between an imbalance in glucose metabolism and the inflammatory response to oxidative stress [38]. IR is known to disrupt lipid homeostasis, which can induce endothelial injury and result in the initiation of atherosclerosis [38–39]. Additionally, IR is thought to be related to increased production of pro-inflammatory cytokines, which can lead to further myocardial injury in established ischemic myocardium, and in turn, increased myocardial oxygen consumption could eventually lead to a reduction in the compensatory capacity in non-infarcted muscles [8, 39]. Therefore, the TyG index could be considered an indicator of residual cardiovascular risk that is further aggravated in STEMI patients with post-PCI progression. Accordingly, the developed prediction nomogram incorporating the TyG index with well-established risk factors may offer increased discriminatory ability and accuracy for STEMI patients after PCI who are at early risk for long-term poor prognosis, and the timely warning provided by this nomogram could in turn inform clinical decision-making and help healthcare providers to allocate resources effectively.

### Study limitations

First, the present study had a small sample size, and thus, it is essential that a large prospective cohort study be conducted to confirm the prediction nomogram’s predictive performance. Second, some heterogeneity in the clinical and demographic data derived from the two centers and incomplete clinical data for some patients could have resulted in some confounding factors being missed that may have affected the conclusions of the study. Third, due to the different methods used by the two centers to measure myocardial injury markers (e.g., troponin), this study did not consider these variables. Fourth, the prediction results from the nomogram were assumed to remain constant over time. However, in reality, disease outcomes may vary due to improved treatment, early detection and changes in its natural history. Therefore, the performance of the nomogram may become less accurate over time.

## Conclusions

A prediction nomogram was developed and externally validated for risk stratification of new-onset STEMI patients with receiving emergency complete and successful revascularization that integrates the TyG index with well-established clinical risk factors. Our data validated the TyG index as a quantitative long-term risk biomarker for identifying STEMI patients at high-risk for poor outcome following PCI. The developed prediction nomogram can be easily applied clinically, facilitating the design of clinical studies of its utility and eventually supporting individual management of STEMI patients. Clinical trials to confirm the benefit of the prediction nomogram and further update its components and cutoff values are warranted.

## Electronic supplementary material

Below is the link to the electronic supplementary material.

Supplementary Material 1

## Declarations

### Data sharing agreement

Individual participant data that underlie the results reported in this article, after de-identification can be obtained from the corresponding author upon reasonable request.
