# Supplementary material for: Predicting long-term prognosis after percutaneous coronary intervention in patients with new onset ST-elevation myocardial infarction: development and external validation of a nomogram model
Source: Cardiovasc Diabetol. 2023 Apr 13;22:87. doi: 10.1186/s12933-023-01820-9 (PMC10103457; doi:10.1186/s12933-023-01820-9)
Supplement: Supplementary file 1 — Supplementary Material 1 [file 12933_2023_1820_MOESM1_ESM.doc]

**Appendix.** **Inclusion and exclusion criteria.**

| **Inclusion criteria** |
| --- |
| New onset ST-elevation myocardial infarction without previously known coronary artery disease (CAD) |
| Complete and successful revascularization administered by percutaneous coronary intervention (PCI) |
| After 1 day after the procedure, glucose levels and lipid levels were measured |
| Follow-up was completed. |
| **Exclusion criteria** |
| Previously known CAD |
| Chronic coronary syndromes |
| Non-ST-elevation acute coronary syndrome |
| Procedure of elective PCI |
| Previously PCI or coronary artery bypass surgery |
| Post-PCI Thrombolysis In Myocardial Infarction flow grade <3 |
| Depressed left ventricular systolic function (ejection fraction <30%) |
| Malignant tumor |
| Atrial septal defect |
| Aortic stenosis |
| Dilated cardiomyopathy |
| Rheumatic heart disease |
| Cor pulmonale |
| Myocarditis or cardiomyopathy |
| Infectious or severe liver or kidney disease |
| Lacked data on fasting triglyceride (TG) and fasting blood-glucose (FBG) |
| Poor compliance to treatment |
| Lost to follow-up. |
